# The Influence of Dietary Interventions on Arterial Stiffness in Overweight and Obese Subjects

**DOI:** 10.3390/nu15061440

**Published:** 2023-03-16

**Authors:** Agata Stanek, Bogna Grygiel-Górniak, Klaudia Brożyna-Tkaczyk, Wojciech Myśliński, Armand Cholewka, Samaneh Zolghadri

**Affiliations:** 1 Department and Clinic of Internal Medicine, Angiology and Physical Medicine, Faculty of Medical Sciences in Zabrze, Medical University of Silesia, Batorego 15 Street, 41-902 Bytom, Poland; 2 Department of Rheumatology, Rehabilitation and Internal Diseases, Poznan University of Medical Sciences, 61-701 Poznań, Poland; 3 Chair and Department of Internal Medicine, Medical University of Lublin, Staszica 16 Street, 20-081 Lublin, Poland; 4 Faculty of Science and Technology, University of Silesia, Bankowa 14 Street, 40-007 Katowice, Poland; 5 Department of Biology, Jahrom Branch, Islamic Azad University, Jahrom 74147-85318, Iran

**Keywords:** overweight, obesity, arterial stiffness, dietary interventions, cardiovascular disease, mortality

## Abstract

Arterial stiffness is often increased in overweight/obese subjects before the development of hypertension. It is also one of the earliest indicators of increased cardiovascular disease risk and can be considered a good predictor of the development of subclinical cardiovascular dysfunction. Arterial stiffness is a significant prognostic factor influencing cardiovascular risk, which dietary habits can modify. Obese patients should use the caloric-restricted diet because it augments aortic distensibility, diminishes pulse wave velocity (PWV), and increases the activity of endothelial nitric oxide synthases. High intake of saturated fatty acids (SFA), trans fats, and cholesterol, typical for the Western diet, impairs endothelial function and raises brachial-ankle PWV. The replacement of SFA with monounsaturated (MUFA) or polyunsaturated fatty acids (PUFA) derived from seafood and plants diminishes the risk of arterial stiffness. The dairy product intake (excluding butter) decreases PWV in the general population. The high-sucrose diet causes toxic hyperglycemia and increases arterial stiffness. Complex carbohydrates with a low glycemic index (including isomaltose) should be recommended to keep vascular health. The high sodium intake (>10 g/day), particularly associated with low potassium consumption, has a deleterious effect on arterial stiffness (↑ baPWV). Since vegetables and fruits are good sources of vitamins and phytochemicals, they should be recommended in patients with high PWV. Thus, the dietary recommendation to prevent arterial stiffness should be similar to the Mediterranean diet, which is rich in dairy products, plant oils, and fish, with a minimal red meat intake and five servings of fruits and vegetables daily.

## 1. Introduction

Currently, obesity is a serious chronic disease, which constitutes not only a health problem but also a social and economic one. The number of overweight and obese subjects is still increasing. This is why the global nature of the obesity epidemic was recognized by the WHO, in 1997. In 2022, the World Health Organization (WHO) reported that there are about 2 billion adults who are overweight, whilst 650 million are obese. It is expected that 2.7 billion adults will be overweight, and over 1 billion will be obese by 2025 if these rates do not slow down [1,2]. 

Both being overweight and obese have a huge impact on the development of many diseases such as nonalcoholic fatty liver, type 2 diabetes, cardiovascular disease (CVD), hypertension and stroke, various forms of cancer, musculoskeletal diseases, chronic kidney disease, sleep apnea, as well as mental health problems. Additionally, overweight and obese subjects are also about three times more likely to be hospitalized for COVID-19. They also have an increased risk of intensive care unit admission, invasive mechanical ventilation, and death [1,2,3,4,5]. 

Additionally, it has been shown that subjects with obesity are likely to have an increase in aortic stiffness, independent of blood pressure level, ethnicity, and age [6]. Moreover, arterial stiffness is often increased in overweight/obese subjects before the development of hypertension [7]. In their study, Kim HL et al. have shown that arterial stiffness may be more strongly associated with abdominal obesity than with overall obesity [8]. It has also been shown that body fat percentage is directly associated with arterial stiffness in long-lived populations, consistent with individuals with lean muscle having more elastic arteries [9]. An increase of two percentage points in the average BMI of society reduces the average life expectancy by one year [10].

Arterial stiffness is an independent risk factor, contributing to the development, progression, and mortality of CVD. It is also one of the earliest indicators of increased CVD risk and can be considered a good predictor of the development of subclinical cardiovascular dysfunction [11,12]. Furthermore, on the one hand, arterial stiffness increases with cardiovascular ageing, but on the other hand, it may be accelerated and occurs earlier in the presence of obesity, insulin resistance, and diabetes [13]. However, increased arterial stiffness is also observed in young people (age 10–24 years) with obesity and obesity-related type 2 diabetes mellitus and is an independent predictor of arterial stiffness even after adjusting for cardiovascular risk factors [14]. Furthermore, the development of vascular stiffness due to obesity is more common in women than in men, and obese and insulin-resistant women lose the protection of the CVD associated with estrogen [15].

Being overweight and obese is related to arterial stiffness and is associated with hypertension. Interestingly, isolated systolic hypertension with high arterial stiffness responds weakly to antihypertensive drugs. As a result, the velocity of the aortic pulse wave (PWV) does not reduce significantly after hypotensive management [16]. A lower response to pharmacological treatment is observed in patients with a high grade of arterial stiffness than in subjects with more distensible arteries. In such cases, non-pharmacological interventions can be beneficial. The study by Dengo et al. has shown that reduced body mass during four months in overweight and obese patients significantly improves aortic and carotid stiffness [17]. Thus, dietary interventions leading to body mass reduction are required to decrease arterial stiffness significantly by improving neuromodulation and blood pressure control [18,19,20].

In this review, we attempt to discuss several aspects related to the association between overweight and obesity and arterial stiffness as well as the influence of body mass reduction and dietary interventions on arterial stiffness on the basis of the most recently published studies.

## 2. The relationship between Obesity, Oxidative Stress, and Inflammation

Obesity is a pathological condition, which is connected with excess calorie intake, and consequent accumulation of free fatty acids (FFA) and carbohydrates. Consequently, the increased oxidative stress markers production occurs via different mechanisms such as activation of protein kinase C (PKC), polyol and hexosamine pathways, and synthesis of superoxide anion [21]. Among oxidative stress markers we distinguish reactive oxygen species (ROS) such as superoxide (O_2_^−^), hydrogen peroxide (H_2_O_2_), and hydroxyl radical (OH−)]. In physiological conditions, oxidative stress markers are neutralized by antioxidants such as superoxide dismutase (SOD) and glutathione peroxidase (GPX) [22]. Among obese subjects, there is an imbalance between antioxidants and oxidative stress markers, which is not only the result of enhanced ROS concentration but also the result of lowered SOD activity [23]. The accumulation of ROS contributes to cell structure damage such as proteins, DNA, and membranes, and to consequent endothelial dysfunction [24]. Moreover, O_2_− reacts with nitric oxide (NO) and consequently decreases its bioavailability. In addition, the product of mentioned reaction (ONOO^−^) promotes the dysfunction of endothelial nitric oxide synthase (eNOS), which causes a further reduction in NO concentration [24]. Increased concentration of FFA contributes to enhanced β-oxidation in mitochondria with a consequent increase in the NADH/NAD+ ratio and increased concentration in advanced glycosylation end products (AGE), and increased activation of PKC, which is responsible for inhibition of eNOS and decreased NO [25]. Increased inflammatory state and oxidative stress are strongly linked with each other and with obesity. Excessive levels of triglycerides and lipids stored in adipocytes among obese patients contribute to hyperplasia and hypertrophy of adipocytes [26]. Enhanced adipocytes induce the elevation of IL-6, IL-8, and leptin and decrease the level of adiponectin, which leads to the consequent accumulation of inflammatory factors in adipose tissue [27]. In addition, macrophages, which are the most numerous immune cells in adipose tissue, consist of two subtypes: M1, with an inflammatory profile, and M2, with an immunosuppressive feature [28]. The pro-inflammatory markers, such as tumor necrosis factor-alpha (TNF-a), and interleukin (IL)-6, secreted from M1 macrophages, induce increased ROS production in adipose tissue of obese patients [29].

## 3. Arterial Stiffness among Overweight and Obese Subjects

**Figure 1:**
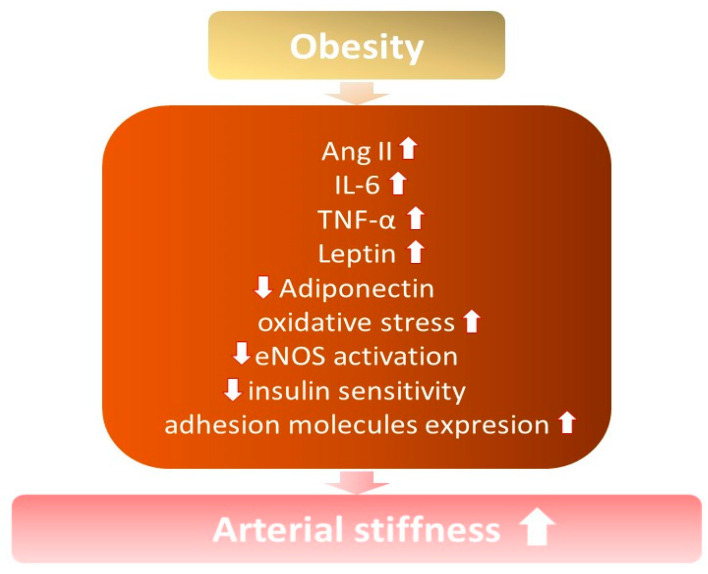
The influence of obesity on the development of increased arterial stiffness. ↓—decrease; ↑ —increase; Ang II—angiotensin II; IL-6—interleukin 6; TNF-α—tumor necrosis factor; eNOS—endothelial nitric oxide synthase.

It is known that arterial stiffness increases among obese and overweight individuals, especially with excess abdominal fat [7,21,22] (Figure 1). Increased arterial stiffness contributes to the development of hypertension, due to structural modifications in the vessels and changes in their elasticity, capacity, and resistance, with a consequent loss of vascular compliance [14].

### 3.1. Inflammatory Mediators

Interleukin 6 (IL-6) and tumor necrosis factor α (TNF-α) are the main inflammatory factors, whose level is elevated among obese patients. IL-6 is produced by different cells such as adipocytes and macrophages, which infiltrate adipose tissue and endothelial cells, while TNF—α is primarily a product of macrophages [23,24]. Both contribute to insulin resistance and impaired insulin metabolic signaling, which is crucial for normal endothelial functioning, which is explained below [25].

IL-6 is involved in the regulation of endothelial function by improving the upregulation of vascular cell adhesion molecule 1 (VCAM-1) and intercellular adhesion molecule 1 (ICAM-1), which have pro-atherogenic facilities [24]. Moreover, IL-6 turned out to be a stimulant of production matrix metalloproteinases, which cause plaque ruptures and changes in arterial vulnerability [26]. Monoclonal antibodies are becoming more and more popular for their therapeutic purposes, especially the IL-6 antagonist, which was widely used during the COVID-19 pandemic [27]. The treatment of atherosclerosis by IL-6 antagonist—tocilizumab—is controversial, while on the one hand, the endothelial function is improved, and arterial stiffness is reduced [28]. On the other hand, the use of tocilizumab in lymphoproliferative disorders causes weight gain and dyslipidemia, which are commonly known as the main risk factors for atherosclerosis [29]. TNF-α, similarly to IL-6, is responsible for the stimulation of VCAM-, ICAM-1, and monocyte chemoattractant protein-1 (MCP-1) production and consequent endothelial dysfunction induced by the synthesis of ROS and inflammatory factors [30]. Treatment with TNF- α antagonist—infliximab—on rodents resulted in a reduction in inflammatory state and protection against insulin resistance and diet-induced obesity [31]. In contrast, studies conducted among participants with insulin resistance did not confirm a positive influence on insulin sensitivity and endothelial function; however, the inflammatory state was reduced [32]. However, obesity is connected with a chronic low-grade inflammatory state, which is confirmed by an increased level of inflammatory markers, especially C-reactive protein, and IL-6, which are significantly higher among obese nonmorbid patients and positively correlate with BMI [33,34].

### 3.2. Hyperinsulinemia 

It is known that obesity, or even overweight, causes impaired insulin release, with hyperinsulinemia, in the beginning, causing the gradual worsening function of β cells of islets of Langerhans. Normal insulin metabolism plays an essential role in maintaining proper endothelial function [18]. Hyperinsulinemia, which is the result of insulin resistance, causes decreased activation of eNOS through an insulin receptor substrate (IRS)-1/phosphatidylinositol 3-kinase (PI3K) signaling/protein kinase B (Akt)-mediated pathway, a decrease in NO bioavailability, and a consequent increase in arterial stiffness [35]. Moreover, there is a correlation between hyperinsulinemia and impaired tissue transglutaminase (TG2) functioning in developing arterial stiffness. Increased TG2 activation induces remodeling and increased resistance in arteries. Additionally, it decreases the bioavailability of NO as a result of impaired S-nitrosylation of TG2 and increased excretion to the cell surface [35].

### 3.3. Renin-Angiotensin-Aldosterone System Activation

Obesity, mainly caused by excess energy intake, especially due to a diet that is not rich in carbohydrates and a high-fructose diet, is associated with increased angiotensin II (ANG II) production by different types of adipose tissue, visceral as well as perivascular adipose tissue (PVAT) [36]. Ang II induces vasoconstriction of vessels and, in increased concentrations, is responsible for the activation of immune cells and the consequent production of cytokines and inflammatory factors [37]. Moreover, increased ANG II causes disturbances in phosphorylation and activation of eNOS through IRS-1/2/PI3K/AKT signaling, which causes decreased production and bioavailability of NO [38]. In addition, Ang II induces enhanced production and release of endothelium-dependent vasoconstrictor, especially endothelin-1 by increased preproendothelin-1, which is consequently changed into endothelin-1, and COX-1-prostanoid by endothelial cells [39,40]. In addition, the role of angiotensin-II type 1 receptors (AT1R) and angiotensin-II type 2 receptors (AT2R) in maintaining proper endothelial function is important. Both receptors are expressed in vascular endothelial cells, while activation of AT1R induces vasoconstriction, and activation of AT2R promotes vasodilation [38]. Persistent activation of AT1R by ANG II induces a decrease in eNOS expression and a consequent decrease in NO concentration. Increased ANG II induces impaired insulin-mediated NO synthesis via up activation of AT1R [38]. Moreover, Ang II, such as aldosterone, increases arterial stiffness by promoting the proliferation of vascular smooth muscle cells (VSMCs), fibrosis, and increased collagen deposition [41].

### 3.4. Adipocyte-Derived Factors

The synthesis and release of factors derived from adipocytes by visceral as well as PVAT are impaired among obese patients. In addition to the factors mentioned above, there is an important change in the production and release of adiponectin and leptin [42,43].

Almabrouk et al. presented their study with mice fed a high-fat diet, and the level of adiponectin was reduced by 70% [44]. There are some data on humans which confirm the point of view that obesity decreases the level of adiponectin, and the relationship between the level of adiponectin and arterial stiffness is inversely proportional [42,43,45]. Furthermore, among obese patients, the peroxisome proliferator-activated receptor gamma (PPAR-γ), which is responsible for the differentiation of adipocytes, is down-regulated, causing a decrease in adiponectin [46]. As a consequence, the anticontractile effect of adiponectin, caused by the activation of adenosine monophosphate-activated protein kinase (AMPK) and consequent phosphorylation of eNOS, is weakened. 

The level of leptin among obese patients is enhanced, while the synthesis of leptin by adipocytes is directly proportional to its size [43]. Leptin has anticontractile properties by increasing NO synthesis; however, prolonged exposure of the endothelium to leptin causes a decrease in NO bioavailability and a consequent inverse effect on vascular tone [47]. In addition, leptin provokes platelet aggregation and atherothrombosis, stimulates ROS synthesis, and causes consequent endothelial dysfunction, resulting in increased arterial stiffness [48,49]. 

## 4. Influence of Body Mass Reduction on Arterial Stiffness in Overweight and Obese Subjects 

The randomized, controlled study conducted on overweight and obese middle-aged and older adults showed that intentional weight loss (hypocaloric diet alone without increases in physical activity) reduces large artery stiffness. This effect is independent of a reduction in abdominal adiposity and is associated with accelerated arterial stiffening and an increased risk of adverse cardiovascular events [17,50,51,52,53]. Thus, body mass fat loss is an independent predictor of arterial stiffness [17]. This effect is observed with modest weight losses of 5% to <10%, associated with a significant decrease in cardiovascular risk in one year. The more considerable weight loss has more significant benefits [50]. For example, modest weight loss markedly improves glycemic control [54], blood pressure [51], and triglycerides [52], and increases HDL cholesterol [53]. The effects of body mass reduction on CVD risk factors are initially the biggest, even if weight losses are maintained for a long time [55].

## 5. The Impact of Diet on Arterial Stiffness

Since arterial stiffness influences vascular ageing and is an independent predictor of CVD and related mortality, the analyses of factors that may decrease its value are a reason for scientific curiosity [56,57]. An increase in vascular stiffness mediates the adverse effects of major cardiovascular risks, including atherosclerosis, hypertension, and diabetes. Thus, preventing arterial stiffness seems reasonable in improving cardiovascular health. In the literature, various nutritional interventions regarding arterial stiffness have recently been widely discussed [58,59,60,61,62,63,64,65,66,67,68,69,70,71,72,73,74,75,76,77,78,79,80,81,82,83,84,85,86,87,88,89,90,91,92,93,94,95,96,97,98,99,100,101,102,103,104,105,106,107,108,109,110,111,112,113,114,115,116,117,118,119,120,121,122,123,124,125,126,127,128,129,130,131,132,133]. The most important include caloric restriction (CR), diets with reduced saturated fat, simple carbohydrates, and sodium intake, and dietary intervention with vitamin supplementation or antioxidative nutrients such as polyphenols. On the contrary, a high-energy and unbalanced diet results in hyperglycemia, elevated non-sterified fatty acid levels, and insulin resistance leading to oxidative stress and inflammation and, consequently, contributing to endothelial dysfunction and the progression of arterial stiffness progression [58].

### 5.1. Caloric Restriction

Arterial stiffness is a significant prognostic factor of CVD, influenced by dietary habits. During the planning of diets, it is crucial to have adequate caloric intake, which improves multiple metabolic parameters and vascular health [59,60,61,62,63,64,65,66]. Ahmet 2011, For example, CR reduces the risk of obesity, diabetes, and CVD and thus increases the lifespan in human and animal models [66]. Food restriction prolongs life in rodents by revealing antiaging effects on various physiologic and pathologic processes [59,60]. CR delays age-related decline in cardiac function, fibrosis, and arterial stiffness [59]. Decreased energy intake prevents increases in arterial stiffness associated with ageing through the inhibition of unbeneficial changes in collagen and elastin [61]. Furthermore, a caloric-restricted diet might influence the ageing process of the arterial wall through various structural alterations in cells and matrix. For example, it delays age-related degenerative features by decreasing the collagenases, increasing the elastin amount, and preserving the vascular smooth muscle in the aorta [60]. Consequently, a larger increase in aortic distensibility and a decrease in PWV are observed [59,60]. Another mechanism present during CR is the possibility of increased activity of endothelial nitric oxide synthases (eNOS). This enzyme increases nitric oxide bioavailability and protects against oxidative stress. As a result, basal NO production increases the distensibility of the large arteries. This mechanism underlines the endothelial role in developing arterial stiffness [62].

Weight loss in overweight and obese individuals is associated with reduced arterial stiffness [63]. Modest weight loss (mean 8% of initial body weight) achieved with diet and lifestyle interventions improves PWV. If weight loss exceeds 10% of initial body weight, it improves the carotid-femoral PWV (cfPWV) of 0.8 m/s [64]. A similar effect of body mass reduction is observed after menopause. The analysis of postmenopausal women obese women has shown that a hypocaloric diet decreases brachial-ankle PWV (baPWV) mainly by reducing femoral-ankle PWV, and this reduction is related to the loss of truncal fat in obese postmenopausal women [65].

Many data showed that caloric CR is strictly related to reduced risk of obesity and decreased arterial stiffness. However, scientific curiosity arises with the question if there is an association between specific dietary components, including macroelements (fat, protein, carbohydrate), microelements, vitamins, and some nutrients, such as polyphenols and carotenoids, on arterial stiffness. All of these nutrients may influence arterial stiffness and change endothelial function. Thus, in this article, the analysis of what kinds of dietary interventions can decrease arterial stiffness and, therefore, improve vascular health and overall cardiovascular risk is analyzed. 

### 5.2. Fat and Fatty Acids Influence Arterial Stiffness 

Several studies underline that both the quantity and quality of fat intake influence the CVD risk [67,68,69] and affect arterial stiffness [69,70,71]. The recommendations of the WHO guideline underline the need to limit fat intake, mainly saturated fatty acids (SFA) and cholesterol, to delay or prevent CVD (Table 1). Simultaneously, increased consumption of unsaturated fatty acids should be considered during diet planning (both polyunsaturated—PUFA and/or monounsaturated fatty acids—MUFA) [72]. Recent data meticulously analyzed the impact of dietary fat quantity and quality on arterial stiffness [69,70,71]. Animal studies showed that high-fat and high-sucrose diets cause the of obesity before or alongside arterial stiffness. In contrast, a normal caloric diet induces weight loss in obese animals and decreases arterial stiffness [63].

One of the leading factors influencing cardiovascular risk is a Western diet, rich in saturated and refined fats, and high-sucrose products [73]. SFA are the most deleterious for vascular health, leading to arterial stiffness. The intake of SFA and trans-fat is associated with a higher risk of CVD [67]. On the contrary, low consumption of SFA for at least two years is associated with a 17% reduction in coronary heart disease events (CHD) [68] and decreases mortality from atherosclerotic vascular diseases in elderly Australian women (*n* = 1469) [69]. The impairment of endothelial function is observed after high-fat meals reach SFA, compared to low-fat meals [71]. A diet containing saturated fat has an unbeneficial influence on vascular health. Such a diet raises brachial-ankle pulse wave velocity (baPWV) in Japanese type 2 diabetic outpatients (*n* = 733) without a history of CVD. On the contrary, lower intake of SFA is correlated with persistently higher baPWV (measured at baseline at 2 and 5 years) [74]. Moreover, decreased amounts of dietary saturated fats by replacing them with polyunsaturated vegetable oil reduced CVD by 30%, and this outcome is similar to the statin-induced reduction [76]. A similar effect is observed if SFA is substituted by unsaturated fatty acids (derived from fish or plants), and such dietary modifications positively influence arterial stiffness [75].

Conversely to SFA, unsaturated fatty acids have a beneficial influence on vascular health. One of the unsaturated fatty acids is MUFA (mainly oleic acid), which is present in nuts, avocados, unrefined (virgin) olive oil, safflower, and canola oil [76]. The replacement of trans fat with MUFAs is inversely associated with CVD [67]. A beneficial effect is also observed if MUFA substitutes for SFA, and this modification causes a lower arterial pulse pressure in diabetic patients (The National Health and Nutrition Examination Survey (NHANES), 2007–2008) [70]. Nevertheless, the exact role of MUFA is not fully defined because some data shows that the replacement of SFAs with MUFA did not cause cardiovascular risk reduction [68].

In addition to MUFA, PUFA intake is also considered beneficial for vascular health. Among PUFA, both n-6 fractions (linoleic acid in vegetable oils) and n-3 fractions (seafood and plant sources) reduce CHD risk [77,78]. The study of Guasch-Ferre et al. showed that the intake of MUFAs and PUFAs in general populations is related to a lower risk of CVD and death [67]. Furthermore, replacing SFA with PUFA results in a 27% reduction in the risk of CHD prevalence [68]. In contrast, the study by Vafeiadou et al. has shown that the substitution of 9.5–9.6% of total energy dietary SFAs with MUFA or n-6 PUFA did not significantly affect the percentage of flow-mediated dilatation. However, such a diet has beneficial effects on biomarkers affecting arterial stiffness, such as E-selectin as serum lipids (fasting serum total cholesterol LDL and CH to HDL ratio) [79].

Many studies have shown that fish consumption has a beneficial influence on myocardial infarction [80] and hypertension [81] and, thus, lower CHD incidence and mortality [82]. The analysis of the Japanese population (*n* = 41,578, aged 40 to 59) who were free of prior diagnosis of CVD showed that high frequency or a large amount of fish intake (eight times per week or 180 g/day) versus lowest (23 g/day) reduced risk of nonfatal coronary events [83]. Fish and fish oils include PUFA, which reveals a beneficial influence on cardiovascular risk. PUFA, mainly linoleic acid (18: 2*n* − 6), and n-3 PUFA have a beneficial effect on vascular function. Among PUFA, the essential fatty acids eicosapentaenoic EPA (20: 5*n* − 3) and *docosahexaenoic* DHA (22: 6*n* − 3) reduce blood pressure and increase endothelium-dependent vasodilation in diabetic and hypertensive patients [71]. Observational studies report decreased arterial stiffness (measured by PWV of the aorta, intima-media thickness of the carotid artery, and atherosclerotic plaques in individuals who habitually eat larger amounts of fish [84]. Some studies suggest that the effect of omega-3 fatty acids may be related to age. For example, 12 weeks of daily omega-3 fatty acids supplementation decreases carotid-femoral PWV in older (66 ± 2-year-old) but not young (25 ± 1-year-old) healthy adults [85]. Moreover, in obesity, the 25% energy deficit diet, including 4 g/d of essential fatty acids—EFA (46% EPA and 38% DHA)—supplementation, improves metabolic parameters and arterial stiffness in obese participants much more than the caloric-restriction diet alone. The changes in systolic blood pressure (SBP), heart rate, plasma TGs, and large and small artery elasticity were significantly greater in the CR with EFA intake. This study shows that supplementation with n-3 EFA improves the elasticity of large and small arteries independently of weight loss in obese adults [86].

One of the most well-analyzed nutritional diets is the Mediterranean one. This diet has received particular scientific interest in recent years as it protects against CVD, particularly compared to a Western diet. The health benefits are related to the balanced composition of macronutrients (carbohydrates, proteins, and fats) [87]. The Mediterranean diet is characterized by a moderate consumption of lean meat and fish with a minimal intake of red or processed meat, minimalizing saturated fat intake [88] This diet habit is associated with a reduction in all-cause and cardiovascular mortality risk caused by decreased inflammation, improvement of endothelial function, and flow-mediated dilatation [89]. Thus, this diet should be recommended in patients with arterial stiffness. 

**Table 1:** Dietary effect of various nutrients on arterial stiffness and cardiovascular risk.

Nutrients Influence Arterial Stiffness			
Component	Dietary Modification	Health Effect/Influence on Arterial Stiffness	References
Fat	high-fat and high-sucrose diet (animal studies)	↑ obesity ↑ arterial stiffness	[64]
	↑ SFA in the diet	↑ arterial stiffness	[67]
	high SFA intake	↑ baPWV at baseline and at 2 and 5 years (diabetic patients)	[74]
	substitution of 9.5–9.6% total energy dietary SFAs with either MUFA or n-6 PUFA	no significant effect on the flow-mediated dilatation beneficial effects on biomarkers of arterial stiffness: ↓E-selectin, ↓ fasting serum lipids (CH. LDL, and CH/HDL ratio)	[79]
	SFA replacement with MUFA and carbohydrates	↓ arterial pulse pressure	[70]
	fish oils consumption with high EPA and DHA in diabetic hypertensive patients	↓ blood pressure ↑ endothelium-dependent vasodilation	[70]
	a habitual large amount of fish consumption	↓ arterial stiffness ↓ pulse wave velocity of the aorta ↓ intima-media thickness of the carotid artery ↓ atherosclerotic plaques	[84]
	the 25% energy deficit diet, including 4 g/d of EFA (46% EPA and 38% DHA) in obese patients	↑ large and small artery elasticity (about 20% increase)	[86]
Protein	vegetarian diet vs. omnivore control	↓ PWV (−8%) in healthy male vegetarians vs. omnivore control sample (7.1 ± 0.8 and 7.7 ± 0.9 m/s, respectively)	[90]
	vegetarian diet vs. omnivore control	a tendency to ↑ arterial stiffness in omnivores (mainly male) vs. vegetarians (PWV 7.0 ± 1.5 vs. 6.8 ± 1.1 m/s, respectively) limited effect in premenopausal women	[91]
	dairy products	inverse correlation with PWV	[92]
	milk consumption	↓SBP (inverse dose-dependent effect) not associated with arterial stiffness	[93]
	dairy products (milk, cheese, cream excluding butter) cohort prospective study (22.8 years observation)	high consumption of dairy products ≥ ↓ 1.8% A. no detrimental to arterial stiffness and metabolic markers (insulin, TG, CH)	[93]
	casein-derived biologically active tripeptides in dairy products	↓ angiotensin formation ≥ ↓ HA risk ≥ ↓ arterial stiffness	[94]
	dairy products containing ruminant-derived fatty acids (14:0, 15:0, 16:0, 17:0, and 17:1)	no influence on serum lipoproteins, PWV, central blood pressure, and AI.	[95]
Carbohydrate	postprandial glucose in non-diabetic patients	↑ postprandial glucose concentration ≥ ↑ arterial stiffness with age (measured by CAVIs) postprandial glycemia is an independent predictor of CAVI values in men and women over 50 years old	[96]
	The high-sucrose diet in diabetic patients with kidney impairments (albuminuria)	postprandial hyperglycemia ≥ ↑ brachial PWV of intermediate-sized arteries (30 min before breakfast and up to 240 min after breakfast)	[94]
	low-carbohydrate diets	↓ serum glucose and lipid level and ↓ aortic stiffness within a short time (four weeks) ≥ ↓ CVD and ↓ diabetes risk	[98,99,100]
	dietary carbohydrate restriction (about 645 kcal/day energy deficit)	↓ body mass, glucose, and lipids ↓ arterial stiffness (↓PWV, significant only in women: from 7.2 ± 03 m/s to 6.3 ± 0.3 m/s, *p* = 0.028)	[100]
	low-GI diet (breakfast) in young, healthy adults compared to high-GI food	↓ arterial stiffness compared to high-GI food high-GI products ≥ ↑ AI and heart rate (but stratifying data by gender, this interaction remained significant for AI only in males)	[101]
	high-GI diet	↑ arterial stiffness immediately after food intake	[96]
	~600 kcal breakfast including fiber with various GI (fiber amount~4 vs. 20 g and GI~44 vs. 70)	high-fiber diet with low-GI ≥ ↑ FMD four hours after meal ingestion	[102]
	A diet containing isomaltulose vs. sucrose glucose	25 g of isomaltulose consumption ≥ stable baPWV in 30, 60, and 90 min after ingestion compared to the state before ingestion in healthy middle-aged and older adults 25 g sucrose intake ≥ ↑ baPWV 25 g glucose ≥ ↑ baPWV at 30, 60, and 90 min after ingestion and ↑ CAVI at 60 min after glucose intake	[103,104]
Elements	sodium intake: low sodium <6 g, medium 6–10 g, and high >10 g sodium daily intake on arterial stiffness.	high sodium intake (>10 g/day) ≥ ↑ arterial stiffness (baPWV ≥1400 cm/s)	[105]
	high sodium in normotensive subjects	AIx	[106]
	↓ sodium intake	↓ blood pressure + ↓ arterial stiffness (independently of antihypertensive effects)	[107]
	low-salt diet	was proved (meta-analysis) ≥ a reduction of 89.3 mmol/day in sodium intake in different populations is associated with a 2.84% reduction in PWV	[108]
	two weeks of dietary sodium restriction in older adults	↓ arterial stiffness (rapid improvement of large elastic artery compliance and AIx) ≥ ↓ systolic hypertension	[109]
	Salt restriction for 6 weeks is untreated in patients with mildly raised blood pressure	↓carotid-femoral PWV ↓ blood pressure ↓urinary albumin and ↓albumin/creatinine ratio	[110]
	diet with an additional 20 or 40 mmol K(+)/d from fruit and vegetables in a group with early-stage hypertension	no change in arterial stiffness and endothelial function	[111]
	potassium chloride and potassium bicarbonate supplementation	↑brachial artery flow-mediated dilatation ↓carotid-femoral PWV	[112]
	low dietary potassium intake in healthy young adults	↑ wave reflection and arterial stiffness ↑ potassium excretion ≥ ↓ aortic AIx + ↓ carotid-femoral PWV	[113]
	low sodium-to-potassium intake ratio	↓ aortic AIx + ↓ PWV	[113]
Vitamins	Vitamin D	↓ central arterial stiffness (about 60.0% m/s)	[114,115,116]
		not affect arterial stiffness short (2–12 months) vitamin D supplementation (1000 IU/day to 120,000 IU/month of cholecalciferol) ≥ no effect on aortic PWV and AIx	[117,118,119]
	Vitamin C	improves flow-mediated dilation ↓ central blood pressure ↓ ADMA—an endogenous eNOS inhibitor ≥ ↑ vascular stiffness	[120,121]
	B vitamins	insufficiency ≥ ↑ homocysteine concentration ≥ endothelial dysfunction ↑ homocysteine + ↑ uric acid levels ≥ ↑ baPWV	[122,123]
phytochemicals	Flavonoids (cranberry juice consumption)	↓ carotid-femoral PWV (immediate but no chronic vasodilatory effect	[124]
	Soy isoflavones	↓ PWV and improving arterial compliance	[125,126,127,128]
		no effect on PWV and AIx	[129,130]
	carotenoids (lycopene intake)	↓ brachial-ankle PWV healthy women ↓ PWV in Korean men	[131,134]
		no effect on arterial stiffness in healthy overweight volunteers	[133]

↓—decrease; ↑—increase; ADMA—asymmetric dimethylarginine; AI—augmentation index; baPWV—brachial-ankle pulse wave velocity; CAVI—cardio-ankle vascular index; CH—total cholesterol; CHD—coronary heart disease; CVD—cardiovascular disease; DHA—docosahexaenoic acid; EFA—essential fatty acids; EPA—eicosapentaenoic acid; GI—glycemic index; FMD—flow-mediated dilation; HA—arterial hypertension; HDL—high density lipoproteins; HOMA—homeostasis model assessment; LDL—low density lipoproteins; MUFA—monounsaturated fatty acids; NHANES—National Nutrition and Health Examination Survey; PUFA—polyunsaturated fatty acids; SBP—systolic blood pressure; SFA—saturated fatty acids.

### 5.3. Protein Intake and Arterial Stiffness 

The primary protein sources in the diet are meat, poultry, fish, eggs, and dairy foods. Meat and eggs contain dietary cholesterol and SFA, which deleteriously affect arterial stiffness (discuss below). 

The beneficial influence of a low-fat meat diet is seen mainly in studies analyzing the impact of diets typical for omnivores and vegetarians/vegans on arterial stiffness, for example, lower PWV (−8%) in healthy male vegetarians (*n* = 44) compared to the omnivore control sample (*n * =  44; 7.1  ±  0.8 and 7.7  ±  0.9 m/s, respectively) [90]. Similarly, the study of Mayra et al. has shown a tendency for higher arterial stiffness in omnivores versus vegetarians (7.0  ±  1.5 and 6.8  ±  1.1 m/s, respectively; *p*  =  0.073), mainly male omnivores (*p*  =  0.006 for the effect of gender and *p* = 0.294 for the effect of the eating pattern effect), suggesting that the beneficial effect of the vegetarian eating pattern may be limited in premenopausal women [91].

Not every type of dietary protein contained in meat reveals a detrimental effect on arterial stiffness. For example, poultry meat is rich in protein and is characterized by low fat content, a high degree of unsaturation of fatty acids, and low cholesterol levels [134]. Its beneficial effect on human health is related to bioactive substances it contains, such as vitamins and antioxidants, and an anticipated proportion of an n-6-to-n-3 ratio close to the recommended ratio of 4:1 [135].

A good source of protein is dairy products. Many studies underline that dairy consumption may prevent or delay the onset of hypertension and is associated with a lower risk of mortality and major CVD events [136,137]. Since decreased arterial stiffness, similarly to lower blood pressure, can be influenced by dairy food intake, modifying nutritional habits can have a protective effect and increase lifespan. The longitudinal study confirmed the beneficial influence of the inverse relationship between dairy consumption and hypertension risk in the adult cohort without arterial hypertension (*n* = 2636 subjects). Over seventeen years of observation, the highest intake of total dairy products and low-fat/fat-free dairy foods were inversely correlated with the values of systolic and diastolic blood pressure (DBP). For yogurt, each additional serving was associated with a 6% reduced risk of incident hypertension [137]. A similar association was reported in urban and rural populations (*n* = 136,384 adults from 21 countries), which were observed over nine years of follow-up. That study proved that dairy consumption was associated with lower mortality risk in CVD events independently of ethnicity [136].

Dairy products not only decrease the risk of cardiovascular events and hypertension but also have a positive influence on arterial stiffness. An inverse relationship between dairy product intake and arterial stiffness measured by PWV pulse wave velocity was proved in the general population [92]. Similar data analyzing dietary habits in the UK population have shown that the highest milk intake was related to a lower SBP compared to the group without milk consumption. Furthermore, the intake of milk and dairy products was not associated with arterial stiffening [93]. Interestingly, the data of the Swedish cohort study (*n* = 103,256 adults) analyzed the effect of various groups of dairy products on vascular health. Surprisingly, this study shows a negative association between dairy product intake and the risk of CVD. It proves that intakes of non-fermented milk (≥ 2.5 times/day) are associated with higher all-cause mortality compared with subjects who consumed milk ≤1 time per week. However, fermented milk and cheese intake is associated with lower all-cause mortality [138].

Some disinclinations in the assessment of dairy product intake on arterial stiffening can be associated with the fat content in these foods. However, the analysis of milk, cheese, cream, and butter on aortic PWV, augmentation index (Alx), and blood pressure did not confirm the detrimental effect of dairy products on CVD risk (except butter). This large cohort prospective study (*n* = 2512 men aged 45 to 59) analyzed cardiovascular health followed up at 5-year intervals for a mean of 22.8 years. The AIx was 1.8% lower in subjects in the highest quartiles of dairy product intake compared with the lowest (*p* trend = 0.021). Consumption of dairy products, excluding butter, was not detrimental to arterial stiffness and metabolic markers (insulin, triacylglycerol, and total cholesterol) [93]. Additionally, another study established that only butter intake could be positively associated with higher all-cause mortality [138]. Moreover, a systematic review and meta-analysis have shown that dairy product consumption has no detrimental effects on arterial stiffness [92]. Another systematic review has shown that the consumption of various forms of dairy products (including high-fat dairy, milk, and yoghurt) leads to favorable or neutral associations with cardiovascular risk (e.g., stroke, CAD, and hypertension) [139].

The confusing data regarding the influence of dairy products on metabolic parameters and endothelial health can result from the numerous non-fat components in dairy fermented and non-fermented products revealing specific nutritional functions and thus explaining various health benefits. Active molecules present in dairy products are biologically active tripeptides derived from casein. They have antihypertensive properties, which probably result from the ability to reduce angiotensin formation. It has been suggested that casein-derived tripeptides may reduce arterial stiffness and improve endothelial function [94]. It is worth underlining that some dairy products also contain ruminant origin 14:0, 15:0, 16:0, 17:0, and 17:1 fatty acids, which do not influence serum lipoproteins, PWV, central blood pressure, or AIx [95]. Thus, planning a diet containing specific protein products, dairy products should be included in daily nourishment, excluding butter. 

### 5.4. Carbohydrates and Arterial Stiffness 

Arterial stiffness is affected by the quantity and quality of ingested carbohydrates, particularly simple sugars, and complex carbohydrates. Moreover, the relationship between arterial stiffness and postprandial blood glucose levels increases with age. This thesis was proved by a large Japanese study (*n* = 1291, age range 23–85 years), which demonstrated that the 1 h postprandial glucose level is associated with increased arterial stiffness (measured by CAVI—cardio-ankle vascular index) in non-diabetic patients. Furthermore, postprandial glycemia was an independent predictor of CAVI values in men and women over 50 years of age [96].

Not only age but also obesity and diabetes mellitus are related to more advanced arterial stiffness, which increases with carbohydrate consumption. The high-sucrose diet has a deleterious effect on vascular health, causing toxic hyperglycemia and predisposing patients to arterial stiffness development. Such changes are mainly observed in diabetic patients with kidney impairments. The analysis of glucose levels in diabetic subjects with albuminuria led to intermediate-sized arteries stiffness. The measurement of glycemia 30 min before breakfast and up to 240 min after breakfast showed that postprandial hyperglycemia increases brachial PWV [97].

On the contrary, low-carbohydrate diets reduce CVD and diabetes risk not only by decreasing serum glucose and lipid level but also by improving aortic stiffness in a short time (four weeks) [98,99,100]. Dietary carbohydrate restriction is crucial in obese patients with insulin resistance and metabolic syndrome. The decreased carbohydrate intake, which causes an energy deficit of about 645 kcal/day, leads to beneficial metabolic changes (reduced body mass, glucose, and lipids). It also diminishes arterial stiffness (reduction in PWV, significant only in women: from 7.2 ± 0.3 m/s to 6.3 ± 0.3 m/s, *p* = 0.028) [100].

Complex carbohydrates and fibers (such as cereals and whole meal pasta) are the main components of dietary products with a low glycemic index (GI). Such foods impact metabolic changes and modulate insulin production [140,141]. Moreover, water-soluble fibers lower the absorption of bile acids and, thus, increase the hepatic conversion of cholesterol into bile acids leading to augmented LDL uptake by the liver [141]. A low-GI diet reduces acute adverse effects on arterial stiffness compared to high-GI food in young, healthy adults (n = 40, consuming breakfast with various GI). The high GI was related to increased AIx and heart rate; however, stratifying data by sex, this interaction remained significant for AIx and increased pressure only in males [101]. Thus, changes in arterial stiffness vary between meals with high and low glycemic indexes. Artery stiffness increases immediately after food intake [96]. Breakfast consumption (~600 kcal), including carbohydrates characterized by various amounts of fiber and glycemic index—GI (fibre amount-4 vs. 20 grammes and GI-44 vs.70,resepctively)—differs from endothelial function. The high-fiber, low-GI diet significantly increased flow-mediated dilation (FMD) four hours after meal ingestion [102]. It is worth mentioning that a low-GI diet can also have a beneficial influence on fetus vascular health in women at risk of gestational diabetes. Low-GI meals were shown to influence the birth weight of the offspring, the length of the birth, and the thickness of the arterial wall in early childhood [142]. 

Not every simple sugar has a deleterious impact on arterial health. Recently, many studies have underlined the crucial effect of isomaltose on vascular health. Isomaltose is a naturally occurring beet disaccharide and can be commercially produced from sucrose on an industrial scale. It is used in various food and drink additives and clinical formula diets as an alternative sugar [143]. Isomaltose breaks down slower than sucrose in the small intestine, which influences glycemia after ingestion. Compared to other low-glycemic sugars (e.g., tagatose or psicose), isomaltulose is unique because it does not escape digestion in the small intestine and is yet a fully available low-glycemic carbohydrate that provides sustained glucose release [103,144]. The study analyzing the influence of this sugar has shown that brachial-ankle PWV (baPWV) did not increase in 30, 60, and 90 min after 25 g of isomaltulose ingestion compared to the state before ingestion; however, it increased after 25 g of sucrose intake (*n* = 10, healthy middle-aged and older adult) [103]. Furthermore, a similar study showed an increase in baPWV at 30, 60, and 90 min after ingestion of 25 g glucose, and an increase in the cardio-ankle vascular index was greater at 60 min than at baseline after ingestion of 25 g glucose [103]. Thus, isomaltulose intake inhibits an acute increase in arterial stiffness compared with the consumption of other carbohydrates, such as sucrose and glucose, which creates a possibility of using this sugar in healthy food production [96,103,144].

### 5.5. The Role of Sodium and Potassium in Arterial Stiffness 

A critical dietary factor modulating arterial stiffness is dietary sodium intake. A high-salt diet leads to hypertension and increases incidences of cardiovascular disease [106,145,146]. An increase in blood pressure is associated with decreased arterial elasticity and thus influences arterial stiffness. This inclination tempts many scientists to analyze the influence of sodium on vascular dilation in various groups of patients. For example, the cohort study of the Chinese population (*n* = 36,324 subjects, aged 49.10 ± 12.57 years) analyzed the influence of various amounts of sodium intake (low sodium <6 g, medium 6–10 g, and high >10 g daily) on arterial stiffness. The high sodium intake (>10 g/day) revealed a deleterious effect on arterial stiffness (baPWV ≥ 1400 cm/s) [105]. Conversely, reduced sodium intake decreases blood pressure and alleviates arterial stiffness. Such changes are observed independently of antihypertensive effects, which suggests that daily salt overconsumption may directly affect arterial stiffness [107]. The beneficial effect of a low-salt diet was proved by a meta-analysis, which showed that an average reduction of 89.3 mmol/day in sodium intake in different populations is associated with a 2.84% reduction in PWV [108]. 

Sodium influences vascular health (increases arterial stiffness) in both the normotensive and hypertensive groups of patients. The data regarding normotensive subjects have shown that high-sodium meal increases the AIx [106] and raises wave reflection and carotid blood pressure after a high-salt diet used for two weeks [145]. In addition, increased salt intake has a deleterious effect on healthy young and middle-aged adults. A seven-day high-sodium diet used by both groups caused an increase in central SBP (measured by radial artery applanation tonometry). The growing SBP can increase wave amplitudes and is one of the crucial mechanisms that regulate arterial pressure and worsen cardiovascular risk [146]. Similar results are observed in older people. Gates et al. have shown that two weeks of dietary sodium restriction influences arterial pressure in older adults, which results in decreased arterial stiffness. In that group of patients, sodium restriction has rapidly improved compliance with the large elastic arteries and AIx, causing a decrease in systolic hypertension [109]. However, Garcia-Ortiz noticed that the relationship between arterial stiffness parameters and carotid intima-media thickness (C-IMT evaluated by ultrasonography) with quartiles of sodium intake resembles a J-shaped curve, illustrating that both very high and low intake ratios correlate with increased arterial stiffness [113]. Decreased salt intake can improve blood pressure in newly diagnosed hypertension. The salt restriction for 6 weeks in whites, blacks, and Asians with untreated mildly raised blood pressure has caused a significant decrease in blood pressure, urinary albumin, albumin/creatinine ratio, and carotid-femoral PWV [110].

Since the blood sodium and potassium concentrations influence blood pressure, the analysis of sodium intake is usually performed in the context of potassium supplementation. The available data regarding the role of potassium in arterial stiffness are inconsistent. Data for high potassium supplementation (with an additional 20 or 40 mmol K(+)/d from fruit and vegetables) do not show changes in arterial stiffness and endothelial function compared to a low-potassium diet used in the early stages of hypertension [111]. Some data indicate that higher potassium excretion can be associated with lower aortic AIx and lower carotid-femoral PWV [113]. However, most studies prove the beneficial effect of adequate potassium intake on vascular and heart health. For example, the study that analyzed potassium chloride and potassium bicarbonate supplementation showed significantly improved endothelial function (measured by flow-mediated dilatation of the brachial artery flow-mediated dilatation) and increased arterial compliance (assessed by carotid-femoral PWV) [112]. The survey by Lennon-Edwards et al. in a group of healthy young adults revealed that low potassium intake in the diet is associated with greater reflection of waves and arterial stiffness. Notably, the beneficial influence of potassium on blood pressure is observed even with high sodium consumption [147]. 

Since both macroelements (sodium and potassium) influence blood pressure and arterial stiffness, some data suggest that their ratio can be used as a reliable marker of vascular health. For example, a cross-sectional study by Garcia-Ortiz et al. showed that the sodium-to-potassium intake ratio is associated with the aortic AIx and arterial stiffness parameters (including PWV) [113]. Some data even report that the sodium-to-potassium ratio is more strongly associated with hypertension and/or SBP and DBP outcomes than either sodium or potassium alone [148,149,150].

The influence of sodium on endothelial function is strictly related to the ability of sodium elimination from urine. The study by Del Giorno has shown that low daytime urinary sodium excretion is associated with increased arterial stiffness and central blood pressure [151]. Analysis of the relationship between urine sodium, urinary sodium to potassium (Na/K) ratio, and arterial stiffness in the group of hypertensive patients without antihypertensive treatment showed that urinary sodium and urinary Na/K ratio are independently related to baPWV [152].

Hypertensive patients using high-sodium and low-potassium diet are encouraged to implement the DASH diet (Dietary Approaches to Stop hypertension). Such diets can also have a beneficial influence on arterial stiffness. DASH diet is rich in vegetables, fruits, and low-fat dairy products, which results in low sodium intake. The DASH diet reduces blood pressure in subjects with and without hypertension, regardless of race and gender. Compared to the control diet, which is typical for the United States, and is characterized by a high sodium level, the DASH diet with a low sodium amount caused a lower mean SBP of 7.1 mmHg in normotensive participants and a decrease of approximately 11.5 mmHg in hypertensive patients with SBP [153]. Therefore, the DASH diet should be recommended for hypertension treatment and prevention. 

### 5.6. Vitamin Intake and Arterial Stiffness

Since supplementation of vitamins can improve endothelial function, many studies recently analyzed the influence of these molecules on vascular health. Intake of ADEC vitamins decreases inflammatory and oxidative stress processes, which are deleterious for endothelial function and arterial wall changes (e.g., collagen deposition, smooth muscle cell proliferation, and elastin fragmentation) [154]. Antioxidative vitamins (C, E, and β-carotene) in fruits and vegetables scavenge free radicals. Furthermore, vitamin C protects the membranes from peroxidation by regenerating their α-tocopherol components [155,156]. Vitamin C influences vascular beds and supports endothelial cells. It scavenges radical species, inhibits apoptosis, and spares endothelial cell-derived nitric oxide helping modulate blood flow. Thus, it prevents endothelial dysfunction, which is the initial sign of arterial stiffness [155]. Ascorbic acid also improves flow-mediated dilation, reduces central blood pressure, and decreases asymmetric dimethylarginine (ADMA). ADMA is an endogenous eNOS inhibitor and vascular toxin that is associated with arterial stiffening [120]. ADMA can cause endothelial dysfunction through eNOS inhibition, raise systemic vascular resistance [157], and increases intima-media thickness [158]. Furthermore, ADMA enhances vascular stiffness and decreases cerebral perfusion in healthy subjects [121]. Thus, vitamin C can benefit arterial stiffness by inhibiting ADMA. 

Recently available data on vitamin influence on endothelial function (strictly related to the stiffness of the artery) is controversial. A meta-analysis by Saz-Lara et al. [114] (22 studies; *n* = 1318 participants in the orally supplemented vitamin group vs. *n* = 1115 subjects in the placebo group) showed no statistically significant effects on arterial stiffness (in both pairwise comparison and frequentist network meta-analysis). However, if oral vitamin supplementation was longer than 12 weeks, vitamin D3 showed a significant reduction in central arterial stiffness, compared with the placebo (−60.0% m/s). Other studies confirm the beneficial effect of vitamin D on cardiovascular risk. Both forms of vitamin D, circulating ergocalciferol (25-hydroxyvitamin) and its active form (1,25-dihydroxy-vitamin D, cholecalciferol), influence endothelial function via endothelial vitamin D receptors (VDR) and enzymes converting circulating vitamin D to the active form. In vitamin D deficiency, an increased risk of CVD and arterial stiffness is observed [115,116]. Vitamin D also reveals antioxidative properties. This function is crucial in the case of exacerbated oxidative and pro-inflammatory processes, which cause vascular injury by increasing the proliferation and migration of vascular smooth muscle cells (VSCM) in vitro [115]. Wu-Wong identified genes regulated by VDR (via DNA microarray technology), which participate in the regulation of human coronary artery VSCM growth and therefore can influence arterial stiffness [116].

However, some data do not confirm the influence of vitamin D on arterial stiffness. Two large meta-analyses have shown that vitamin D supplementation is associated with improved endothelial function but does not affect arterial stiffness [117,118]. Similarly, a systematic review and meta-analysis of Upala et al. have shown that short implementation of vitamin D (from 1000  IU/day to 120,000  IU/month of cholecalciferol) for 2 to 12 months does not improve aortic PWV and AIx [119].

The effect of B vitamins on arterial stiffness is mediated by their influence on homocysteine metabolism, which requires the proper intake of folic acid contents, B12, vitamin B6, and riboflavin. Insufficiency of these vitamins increases the concentration of homocysteine, a molecule that causes endothelial dysfunction [122]. The 10-year analysis of cardiovascular risk in the general population has shown that homocysteine activity revealed its mutual effect with uric acid on arterial stiffness. This analysis proved that high levels of homocysteine and uric acid are associated with the highest baPWV compared to the group characterized by low levels of homocysteine and low levels of uric acid (UA) [123].

Recent data confirm the hypothesis that arterial stiffness is one of the possible pathways between hyperuricemia and cardiovascular disease. High serum UA concentrations impair the generation of vasoactive mediators in endothelial cells, including nitric oxide (NO) [159,160]. The decreased NO bioavailability promotes endothelial dysfunction, increases vascular tone, and contributes to arterial stiffness [161]. A decrease in serum nitric oxide rises after lowering UA levels [162]. UA also causes vasoconstrictive dysregulation by inducing oxidative stress. Pro-oxidant molecules stimulate endothelin-1 synthesis, a potent vasoconstrictor that increases arterial stiffness [163]. Besides this, UA stimulates VSMCs proliferation and ROS by stimulating the vascular renin-angiotensin system (RAS) [161]. RAS is responsible for increased stiffness of the cytoskeleton in endothelial cells and extracellular matrix fibrosis [164,165]. Subsequently, vascular hypertrophy, impaired NO synthesis, and arterial stiffness is observed [162,165,166]. Thus, high UA levels stimulate oxidative stress, induce endothelial VSMCs proliferation, and increase endothelin level leading to vascular dysfunction [162]. Effective treatment to prevent or reduce serum UA concentration should be implemented in each hyperuricemic patient to decrease ROS generation, prevent endothelial dysfunction, and reduce arterial stiffening. 

Recent data also underline the potential roles of folate and/or related B vitamins in protecting against CVD (especially stroke), certain cancers, cognitive impairment, and osteoporosis [167]. Nicotinic acid (niacin) is known for its lipid-lowering properties and thus the reduction in myocardial infarction, stroke, and atherosclerosis. However, this vitamin also has anti-inflammatory and potentially anti-atherosclerotic properties independent of its effects on lipid regulation. For example, niacin inhibits vascular inflammation by decreasing endothelial ROS production and subsequent LDL oxidation. As a result, it decreases inflammatory cytokine production in cultured human aortic endothelial cells [168].

The intake of fruits and vegetables has a beneficial influence on arterial stiffness and supports the recommendation to consume more than five servings of fruits and vegetables per day, reducing cardiovascular risk, which is partially related to vitamin consumption [153]. The protective effects of fruits and vegetables are related to their antioxidant properties, which result from a high concentration of antioxidant vitamins, minerals, and metabolically active vitamins (e.g., vitamins from group B) [169]. Therefore, products containing specific vitamins should be included in the daily diet (Table 2).

### 5.7. Phytochemicals and Arterial Stiffness

Phytochemicals include a large group of active compounds with antioxidative, antiproliferative, anti-inflammatory, and inhibitory angiogenesis molecules important in preventing chronic diseases. They reduce neointimal thickening by inhibiting smooth muscle cell proliferation, thus improving endothelium-dependent vasorelaxation. Phytochemicals also modulate the bioavailability of nitric oxide and voltage-gated ion channels [178]. The main phytochemicals groups are polyphenols, flavonoids, isoflavonoids, anthocyanidins, phytoestrogens, terpenoids, carotenoids, limonoids, phytosterols, glucosinolates, and fibers [172].

Polyphenols are the natural components of various fruits, vegetables, cereals, and beverages, which may reduce arterial stiffness. About 100 g of fresh grapes, apples, pears, cherries, and berries deliver up to 200–300 mg of polyphenols. They are also present in red wine, tea, and coffee and in smaller amounts in cereals, dry legumes, and chocolate [173,174]. Flavonoids are the most abundant polyphenols in the human diet [176]. Flavonoids (flavonols, flavones, and isoflavones) have antioxidative, anti-inflammatory, and antithrombotic properties. Plant food, including apples, berries, grapes, and onions, are the main dietary sources of these molecules [176,177,182]. Flavonoids inhibit lipid peroxidation, promoting vascular relaxation and preventing atherosclerosis. In the study by Dohadwala et al., chronic consumption of cranberry juice (including a high dose of polyphenols) reduced carotid-femoral PWV; however, only an immediate effect of such consumption was observed and without chronic influence on vasodilator endothelial function [124]. 

Other nutrients that seem to benefit arterial stiffness are phytoestrogens [125,183]. Phytoestrogens (isoflavonoids genistein, daidzein), flavonoids, and anthocyanins bind to estrogen receptors in physiological concentrations, increasing eNOS activity and NO synthesis [184]. Phytoestrogens, such as isoflavones and lignans, benefit vascular beds and reduce PWV [128]. Soy isoflavones induced nitrite/nitrate levels, decreased endothelin (ET-1) levels, and alleviated arterial stiffness in men and postmenopausal women [178]. Therefore, diets with a high intake of soy isoflavones benefit arterial stiffness by reducing PWV and improving arterial compliance. All these effects can partially explain the decreed CVD risk in populations with a high soy intake [125]. Furthermore, isoflavone intake shows improved arterial stiffness (decreased PWV) [126,127]. Still, some studies did not confirm such influence (no effect on PWV and AIx) [129,130]. Differences in these studies can result from isoflavone intake (different forms and amounts) and specific populations (characterized by various ages, sex, cardiovascular risk, and comorbidities).

Similarly to flavonoids, beneficial antioxidative effects have carotenoids. They reveal antiatherogenic properties by reducing inflammation and ROS formation within the arterial wall. Carotenoids with many conjugated double bonds demonstrate their antioxidant potential. They also improve endothelial function [179] and decrease arterial stiffness [180]. One of the most popular carotenoids is lycopene, found in tomatoes, watermelon, papaya, red grapefruits, and guava. Its antioxidant properties are very high among carotenoids [180]. Lycopene also effectively reduces cardiovascular risk, making it one of the most important dietary components in the daily diet of the general population [181]. An inverse relationship between circulating lycopene and brachial-ankle PWV was observed in healthy women (*n* = 264, 31–75 years), which showed a significant decrease in baPWV with increased lycopene concentration [131]. A similar inverse correlation was found between PWV and serum lycopene in Korean men (*n* = 299) after considering confounding factors such as blood pressure, insulin resistance, and oxidative stress [132]. On the contrary, the study of healthy overweight volunteers (*n* = 255, aged 40–65 years) showed that a relatively high daily consumption of tomato products (32–50 mg lycopene/day) or lycopene supplements (10 mg/day) was ineffective in reducing conventional cardiovascular risk markers and arterial stiffness in middle-aged individuals [133]. Thus, the lycopene effect can vary between the analyzed population, body fat content, and related diseases. 

## 6. Dietary Recommendations to Prevent Arterial Stiffness 

Since an ageing society characterizes the general population, it is crucial to control the progression of arterial stiffness using a well-balanced diet. Such a diet should contain adequate energy intake to maintain proper body mass. In the case of overweight and obesity, caloric restriction should be considered. A well-balanced diet is one of the most important recommendations for preventing arterial stiffens. Such a diet should contain a proper quantity and quality of protein, fat, and carbohydrate (Table 2). 

Two diets can have beneficial effects on vascular health—the DASH diet and the Mediterranean diet. Both diets are rich in vegetables, fruits, low-fat dairy products, and minimal sodium intake. They are widely recommended to diminish or prevent hypertension and cardiovascular events and minimize the risk of arterial stiffness; furthermore, these types of diets decrease inflammation, improve endothelial function, and increase flow-mediated dilatation [89,153,175].

**Figure 2:**
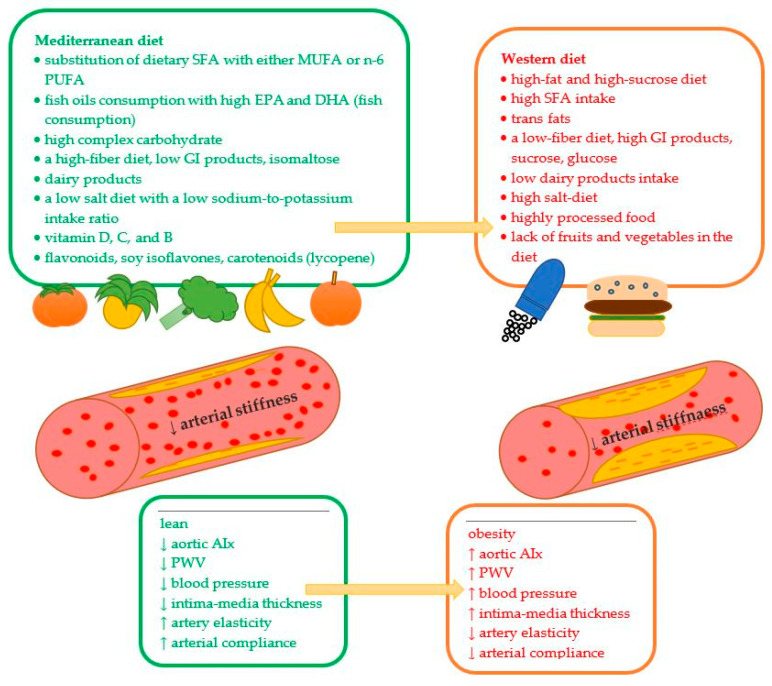
The influence of selective nutrients on arterial stiffness.↓—decrease; ↑—increase; AIx—augmentation index; PWV—brachial-ankle pulse wave velocity; DHA—docosahexaenoic acid; EFA—essential fatty acids; EPA—eicosapentaenoic acid; GI—glycemic index; MUFA—monounsaturated fatty acids; PUFA—polyunsaturated fatty acids; SFA—saturated fatty acids.

Adequate fat intake should include a low amount of trans and saturated fats, possibly replacing them with MUFA and PUFA (Figure 2). Fermented dairy products (without butter), fish, and lean meat are good protein products that can decrease arterial stiffness. Since low glycemic index products benefit postprandial vascular responses, they should be considered when planning daily meals. Isomaltose (bee sugar) can be used to sweeten some meals because it does not have a deleterious effect on postprandial hyperglycemia. Reducing excessive salt intake in the diet is important for overall vascular health and blood pressure. Products including potassium and low sodium: potassium intake ratio is required to decrease blood pressure and reduce arterial stiffness. Active antioxidative, antiproliferative, and anti-inflammatory elements of the diet, such as vitamins and phytochemicals, are crucial for the prevention of chronic diseases and can protect against atherosclerosis and arterial degeneration through an effect on arterial walls.

## 7. Conclusions

Since endothelial dysfunction and increased arterial stiffness are predictors of CVD, a well-balanced diet should be the first step to maintaining vascular health. This review gathers the main dietary recommendations supported by multiple studies. It underlines the role of nutritional habits in overweight or obesity and suggests healthy behavior which can help reduce body mass and improve endothelial function. Striving for a correct body weight through a negative energy balance should be the first element of prophylaxis in excessive body mass and arterial stiffness prevention. Low-fat and high-fiber products (low-energy and food characterized by low GI index) in the diet are crucial for proper body mass achievement. 

Unfortunately, the Western diet (rich in energy overconsumption and a large amount of SFA, trans fats, red meat, simple sugars, and sodium) is the leading cause of obesity and arterial stiffness. High-fat and high-sucrose diets typical for quickly available food not only increase body mass but also have harmful effects on vascular health. Replacing red meat with fish, margarine with olive oils, and sweets with vegetables can be the first step in decreasing adiposity. In addition, normalization of the metabolic state by weight loss returns arterial stiffness and blood pressure to normal. Patients (independently of co-existed diseases) should know the basic dietary recommendations to maintain general and vascular health. The role of nutritional education, in this case, is invaluable. Heightened awareness of the adverse consequences of obesity and obesity-related complications (hypertension, diabetes mellitus) should prompt healthcare providers to institute early and aggressive lifestyle interventions in overweight and obese patients. Thus, there is a need for the development of early detection and prevention programs to mitigate this potentially serious health problem.

Future research should examine arterial stiffness and its influence on cardiometabolic health outcomes in various populations characterized by a specific age, body mass (overweight and obesity), and co-morbidities (diabetes mellitus, cardiac events, metabolic syndrome). Such data can provide valuable insights into the role of particular nutrients and eating patterns on arterial stiffness and cardiometabolic health. The not-fully defined role of specific products in arterial stiffness (e.g., milk, non-fermented products, potassium-rich meals) should be carefully analyzed. Considering the epidemic incidence of obesity worldwide, caused mainly due to excessive consumption of a high-energy diet, arterial stiffness could represent a novel therapeutic target to prevent obesity-associated cardiovascular complications. Początek formularza.

## Figures and Tables

**Table 2:** Dietary recommendation to decrease cardiovascular risk and arterial stiffness.

Dietary Modification in the Prevention of CVD and High Arterial Stiffening			
Modifications	Dietary Recommendations	Effect	Reference
Keep isoenergetic diet	Keep a balance of energy intake no additional meals and snacks	↓ weight loss ↓ arterial stiffness	[63]
↓ SFA intake and cholesterol ↑ MUFA and PUFA in the diet	↓ fat meat, eggs, sweat intake minimize the intake of red meat and replace it with poultry and fish) use Mediterranean diet	↓ myocardial infarction ↓ hypertension ↓ CHD incidence and mortality ↓ risk of nonfatal coronary events ↓ CVD risk	[67,72,80,81,82,83,88,134,135,140]
SFA replaced by MUFA and PUFA	Replace fatty red meat with lean meat, olive oil, fish, dairy products, nuts, and seeds Mediterranean diet	↓ SFA intake at least two years ≥ 17% CHD events reduction ↓ atherosclerotic vascular disease ↓ mortality (mainly in the elderly) SFA replacement by MUFA—no effect on ↓ CVD risk PUFA (vegetable oil) ≥ ↓ CVD by ≈ 30% (similar to the statin effect)	[68,69,73,170]
avoid trans fats or replace them with MUFA	Eliminate or minimize consumption of margarine, fried potatoes, potato chips, corn chips, popcorn, animal products, household shortening, cakes, cookies, crackers, candy	↓ CVD risk	[67,171]
include dairy products	Intake of milk, cheese, and cream (particularly fermented products such as whey, buttermilk, natural yogurt, sweetened yogurt, and matured/semi-matured cheese) Non-fermented milk consumption is not necessary Avoid high-fat dairy such as butter Mediterranean diet	↓prevent or delay the onset of hypertension (both SBP and DBP) ↓ major CVD events ↓ CVD mortality independently of ethnicity each additional serving of yogurt ≥ ↓ 6% risk of HA incident active tripeptides ≥ ↓ angiotensin formation ≥ ↓ HA risk ≥ ↓ arterial stiffness non-fermented milk (≥2.5 times/day vs. ≤1 time per week) can ↑ all-cause mortality fermented milk and cheese intakes ≥ ↓all-cause mortality butter intake ≥ ↑ higher all-cause mortality	[88,93,136,137,138,139]
eliminate simple carbohydrates	Avoid sweets (glucose, sucrose)	Simple sugars ↑ fasting and postprandial glucose concentration ↑ CAVI values with age, particularly > 50.	[96]
use a diet with a low GI	Implement in the diet complex carbohydrates and fibers (cereals and whole-meal pasta)	impact metabolic changes and modulate insulin production ↓ absorption of bile acids ≥ ↑ hepatic CH conversion into bile acids leading to ↑ LDL uptake by the liver	[140,141]
avoid sugary drinks	For sweating, use isomaltose Instead of sweets, use isomaltose-containing products, e.g., food and drink additives or clinical formula diets Mediterranean diet	isomaltose sustained glucose release inhibits an acute ↑ arterial stiffness compared to sucrose and glucose	[88,96,103,143,144]
eliminate or minimalize sodium intake	Decrease salt intake (even for a short time, e.g., 7 to 14 days)	High-salt intake ↑hypertension ≥ ↓ arterial elasticity + ↑ arterial stiffness ↑ incidences of CVD after 7 days of intake ↑ wave reflection and carotid blood pressure after 14 days of intake	[106,145,146]
↑products rich in potassium	Dried fruits (raisins, apricots), beans, lentils, potatoes, pumpkin, spinach, broccoli, beet greens, avocado, bananas	↓ blood pressure (even with high sodium consumption)	[147]
keep a low Na: K ratio in the diet	Avoid kitchen salt Eat products rich in potassium	high Na:K ratio ≥ higher ↑ SBP and DBP than either sodium or potassium alone	[148,149,150]
use DASH or the Mediterranean diet for hypertension	DASH diet: a low sodium diet rich in vegetables, fruits, and low-fat dairy products Mediterranean diet—rich in vegetables, seeds, legumes (e.g., lentils and beans), fruit, cereals, and whole grains (e.g., unprocessed maize, millet, oats, wheat, and brown rice), containing lean meat, fish, and olive oil	↓ blood pressure in subjects with and without hypertension, regardless of race and gender	[88,153]
antioxidative vitamins (C, E, and β-carotene)	Fruits and vegetables (minimum five servings daily)	scavenge free radicals vitamin C protects membranes from peroxidation by regenerating α-tocopherol, scavenges radical species, prevents endothelial dysfunction, ↓ CVD risk	[153,155,156]
B vitamins	Lean meat, fish, milk, cheese, eggs, some fortified breakfast cereals	↓homocysteine level folate prevents stroke, cancers, cognitive impairment, and osteoporosis Nicotinic acid (niacin) ≥ hypolipemic effect + ↓endothelial ROS synthesis ≥ ↓LDL oxidation ≥ ↓inflammatory cytokine synthesis in aortic endothelial cells	[122,123,169]
include products with polyphenols, tocopherols, and phytosterols	Eat fresh red grapes, apples, pears, cherries, and berries cereals, dry legumes, whole grains, cereals, peanuts low amount of red wine, tea, coffee, and chocolate Mediterranean diet	↑ polyphenols, anti-inflammatory effect protect against CVD ↓ overall mortality	[73,89,172,173,174,175]
increase intake of flavonoids	Plant food, including apples, berries, grapes, and onions	inhibit lipid peroxidation ≥ promote vascular relaxation and prevent atherosclerosis Chronic consumption of cranberry juice (including a high dose of polyphenols) ≥ ↓ carotid-femoral PWV (immediate but without chronic vasodilatory effect)	[124,172,173,174,176,177]
implement soy isoflavones	Soy and soy products Other isoflavones: fruits, vegetables, cereals, beverages, legumes, chocolates, oilseeds	↑ nitrite/nitrate levels ↓ ET-1 levels alleviated arterial stiffness in men and postmenopausal women	[172,173,178]
add carotenoids to the diet	Tomatoes, watermelon, papaya, red grapefruits, guava, carrots, parsley, orange and green leafy vegetables, chenopods, fenugreek, spinach, cabbage, radish, turnips	antiatherogenic properties ↓ inflammation and ROS formation within the arterial wall lycopene: high antioxidant properties,↓ CVD risk	[172,174,179,180,181]

↑—increase; ↓—decrease; CAVI—cardio-ankle vascular index; CH—total cholesterol; CHD—coronary heart disease; CVD—cardiovascular disease; DASH—Dietary Approaches to Stop hypertension; DBP—diastolic blood pressure; ET-1—endothelin-1; GI—glycemic index; HA—arterial hypertension; HDL—high density lipoproteins; LDL—low density lipoproteins; MUFA—monounsaturated fatty acids; PUFA—polyunsaturated fatty acids; ROS – reactive oxygen species; SBP—systolic blood pressure; SFA—saturated fatty acids.

## Data Availability

We used PubMed and Web of Science to screen articles for this narrative review. We did not report any data.
